# Occlusion, with Retention of Menses

**Published:** 1874-11-15

**Authors:** W. H. Byford


MEDICAL EXAMINER.

A

Semi-Monthly Journal of Medical Sciences.

EDITED BY N. S. DAVIS, M.D., AND F. H. DAVIS, M.D.

No. XXII. CHICAGO, NOV. 15, 1874. VOL. XV.

### Original Communications.

### OCCLUSION, WITH RETENTION OF MENSES.

REPORT OF A CLINICAL LECTURE DELIVERED IN MERCY HOSPITAL, BY PROF. W. H. BYFORD, M. D.

*Reported by W. H. Warn, M. D.*

TO-DAY, gentlemen, I bring before you this girl, on whom I operated some weeks since for occlusion, with retained menses. The following is her history: M. O———, of M———, Wis., aged sixteen. When she was six years old she had a severe attack of measles, or possibly scarlet fever, and, during convalescence, suffered from inflammation of the middle ear, on both sides, which resulted in partial deafness. During her fourteenth year she began to show signs of menstruation, such as, at irregular intervals, nervous irritability, weight and fullness in the pelvis. For more than a year past these symptoms, together with cramps through the hypogastric region, have occurred at regular intervals of about four weeks, lasting a week, and then followed by another period of rest.

External examination showed a soft, fluctuating tumor in the pelvis, about the size of a uterus at the end of a five months' pregnancy. In making a digital examination the vagina was found to terminate in a cul-de-sac. The patient having been anæsthetized, a trocar was introduced into the fluctuating tumor at the internal extremity of the cul-de-sac, and on removing it, a thick, dark brown fluid flowed slowly out through the canula. The opening was then enlarged, and altogether about a quart of menstrual fluid was evacuated. After washing out the cavity with warm water the patient was carried into the ward and absolute rest enjoined. The opening was prevented from closing up by the introduction of the finger each day. No unfavorable symptoms occurred during the recovery, except on the second night she was seized with sudden and severe pain in the left iliac region, which the house physician, Dr. Haines, promptly relieved with opiates.

Three weeks after the operation she menstruated naturally, the flow lasting about forty-eight hours.*

Occlusion, with retained menses, may be, with rare exceptions, classed under four forms:

1st. An imperforate hymen, oftentimes as much as a fourth of an inch in thickness, and so dense and strong as not to be ruptured without surgical aid.

2d. Absence of vagina. All of the external organs of generation are complete and normal, but the entire vagina is wanting, and in its place, between rectum and bladder, is found a dense wall of tissue.

3d. Occlusion of the os uteri.

4th. Obliteration of a portion, or the whole of the vaginal canal.

*Causes.*—The first and second forms of occlusion are congenital, while the two latter are generally acquired. A frequent cause of acquired occlusion is inflammation and ulceration of the lining membrane of canals and cavities, which take place occasionally during an attack of some of the eruptive fevers.

You will notice in the history of this case which you have just heard, that at the age of six she suffered from a severe attack of an eruptive fever; that there was at this time inflammation of the middle ear, followed by partial deafness. Probably at the same time similar pathological changes took place in the lining structure of the vagina. It became denuded of its epithelium, and the underlying tissue participating in the inflammatory process, union of the opposing surfaces took place, either by first intention or granulation. She being only six years of age there was nothing to call attention to this fact, and it only became apparent after she began to menstruate. The cramps in the uterine region which occurred in this case at the menstrual periods for the past year were caused by the effused blood from the lining membrane of the uterus, acting as a foreign substance, and provoking the uterus to expel its contents. I was asked the other day at the time of the operation, why the effused blood was not absorbed. Undoubtedly a large portion of the blood which was poured into the uterine cavity was absorbed, viz.: the serum, leaving behind the thick, tenacious fluid, which has been found, on microscopical examination, to consist principally of blood corpuscles and epithelian debris.

But the most common cause of vaginal inflammation leading to occlusion, is prolonged and difficult parturition, especially if there has been negligence and want of care after the labor.

Criminal abortion might be mentioned in this connection; and, as an illustration from such a cause, is the case of the young woman on whom I operated in this hospital during the past winter. The principal points of interest in the history of the case were as follows: Menstruation was naturally and completely performed as early as in her fourteenth year. When she had nearly reached the age of sixteen she had a severe and prolonged at-

* The patient was heard from, through the family physician, in October. She had menstruated normally since returning home.

tack of typhoid fever, which left her weak and prostrate. During her illness, and for some-time afterwards, she had a discharge from the vagina. To what extent, if any, the walls of the vagina were at this time injured, I am unable to state; at all events, soon after her complete recovery she became pregnant. During the early months of her pregnancy she procured the services of an irregular physician, who produced an abortion, from the effects of which, after a long sickness, she barely escaped with her life. From this event till the time she came into the hospital, a period of two years, she saw no external signs of menstruation. Physical examination revealed a soft tumor the size of a child's head in the pelvis, and the vagina to end in a cul-de-sac some three or four inches long. After an opening had been made through the obstruction and a quantity of thick, tenacious fluid evacuated, I examined carefully the parts and found that the obstruction was situated at the internal extremity of the vagina, leaving a space of about an inch in length between the obstruction and the mouth of the womb. The after-treatment in this case was in every respect identical with that of the patient before you to-day, but, I am sorry to say, it terminated fatally on the third day, with all the symptoms of acute toxæmia.

I suppose that after the abortion the vagina took part in the inflammatory process, which occurred in the uterine region, and through want of proper care the walls of the vagina, at its upper portion, were allowed to become glued together, and finally firmly united.

Another, though less frequent cause, is extensive and continued syphilitic and gonorrhœal ulceration along the genital tract. Plastic lymph is thrown out, it becomes organized and contracts into dense cicatrical tissue; and when the process continues for a long time a complete and imperforate obstruction is liable to be built up. Several cases have been reported in which the occlusion was the result of traumatic injury. Such a cause is not of frequent occurrence.

*Diagnosis.*—It is rarely ever that the physician is consulted in these cases until the retained menses have caused considerable annoyance and anxiety. The history of the case, then, will go far towards making up a diagnosis, and will lead to a physical examination. External manipulation will show that the uterus is enlarged, and can be felt as a soft, fluctuating tumor, low down in the pelvis. If you introduce a catheter into the bladder you will notice that it takes a direction directly upwards instead of backwards. Now, if at the same time you introduce a finger into the rectum, there will be readily felt between the two an elastic tumor, in size proportionate to the time which the menses have been retained. On attempting to introduce the finger into the vagina it will meet with and recognize the obstruction.

*Treatment.*—The removal of retained menses is always attended with danger, and not unfrequently followed by death; indeed, several cases have been reported where a simple puncture of the imperforate hymen has terminated fatally. All authors agree as to the removal of the fluid, but as to the best method of doing it there are various opinions. The method I have pursued is as follows:

If it be a case of imperforate hymen, I catch up on the point of a tenaculum the central part of the hymen, and, with a pair of scissors, snip out a portion, leaving a small round opening. Then, radiating from the margin of this opening, I make several incisions: thus finally leaving it star-shaped, in imitation of the carunculæ myrtiformes, the remains of the hymen when ruptured without surgical aid. In all other forms of occlusion I puncture the obstructing tissue with a trocar and canula, such as is ordinarily used for tapping in ascites. After withdrawing the trocar I insert through the canula a gum-elastic catheter, to be left as a guide in finding the opening after the canula has been removed. Then with a bistoury I enlarge the opening till it will readily admit the finger. The retained fluid which before flowed slowly through the canula will now flow out readily through the opening, and with gentle pressure over the uterus its contents will be quickly evacuated. The next step is to wash out the cavity with warm water, by the means of a syringe, till the water returns clear. Some authors advise you to dissect through the obstructing tissue with the knife or scissors, in a direction towards the os uteri. The difficulty experienced in endeavoring to dissect this part, with no reliable guide as to the direction in which the dissection is being made, can only be appreciated after making the attempt. In several cases which have come to me from the country, I have noticed that this plan of operating has been tried and in every case failed. Just here I will allude to a case I have in mind on which I operated several years ago. There was complete absence of the vagina and retention of menses, during a period of about two years. The plan of operating was the same as pursued in the case of this girl before you. At the end of three months the entire length of the artificial canal was healed. Five years ago she married, and since then has given birth to one child. She writes that the labor lasted twelve hours, when the child was delivered with the aid of forceps. Her recovery from the confinement was rapid, and since, her health has been good. Again, opinion is about equally divided as to whether a large opening should be made and the retained fluid evacuated at once, and afterwards the cavity washed out, or that the opening should be small and the fluid allowed to escape by drops. It is claimed by the advocates of the latter mode of operating that the "tar-like" fluid cannot be thoroughly washed out, and hence decomposition and septicæmia is as likely to take place whichever plan is pursued. In the cases which have come under my observation, I am confident that all the menstrual fluid has been completely washed out; neither have I ever seen anything in the discharge after the operation which led me to believe that any part of it was some of the retained fluid. I have operated in hospital and private practice in six cases of occlusion: two from absence of the vagina, two from imperforate hymen, two from obliteration of a portion of the vagina, and of the number only one has terminated fatally. This result, and the reports of cases which have been treated in various ways, seems to me to indicate that the opening should always be made sufficiently large to allow the retained menses to be evacuated at once, and the cavity afterward to be washed out.

*After-treatment.*—There is always a tendency in the artificial canal or opening to close up. To prevent this, a plug of lint soaked in glycerine, or a glass plug, should be inserted. What I have found to be quite as efficient is simply to introduce the finger once a day till the parts are completely healed.

